# Supplementary figures and images for: Rational social distancing in epidemics with uncertain vaccination timing
Source: PLoS One. 2023 Jul 21;18(7):e0288963. doi: 10.1371/journal.pone.0288963 (PMC10361534; doi:10.1371/journal.pone.0288963)

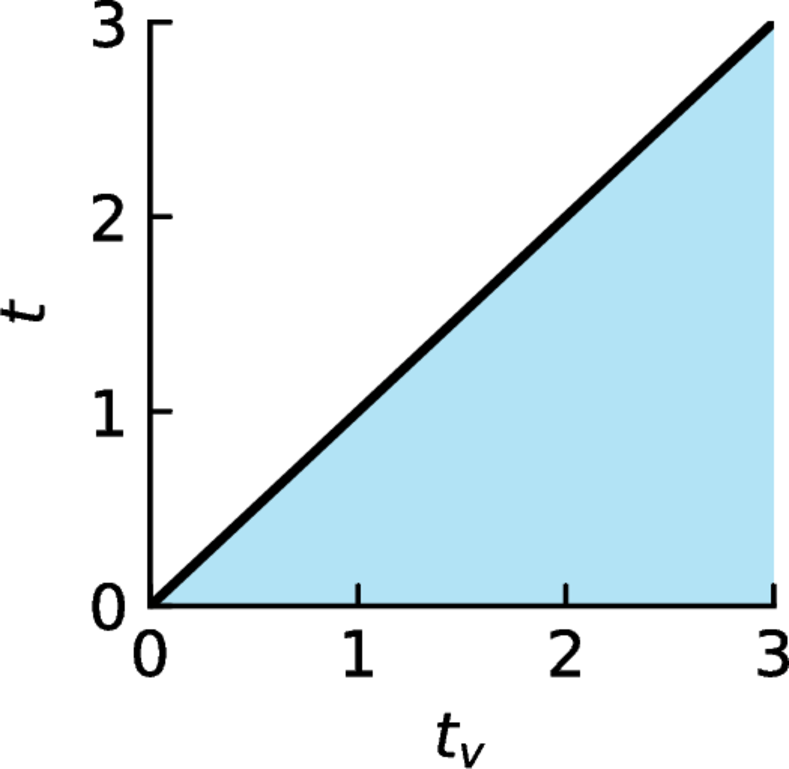

Supplement: S1 Fig — The integration area is marked in blue. For each fixed tv, we integrate t from 0 to tv. Alternatively, for fixed t, we can integrate tv from t to ∞. (TIF) [file pone.0288963.s001.tif]

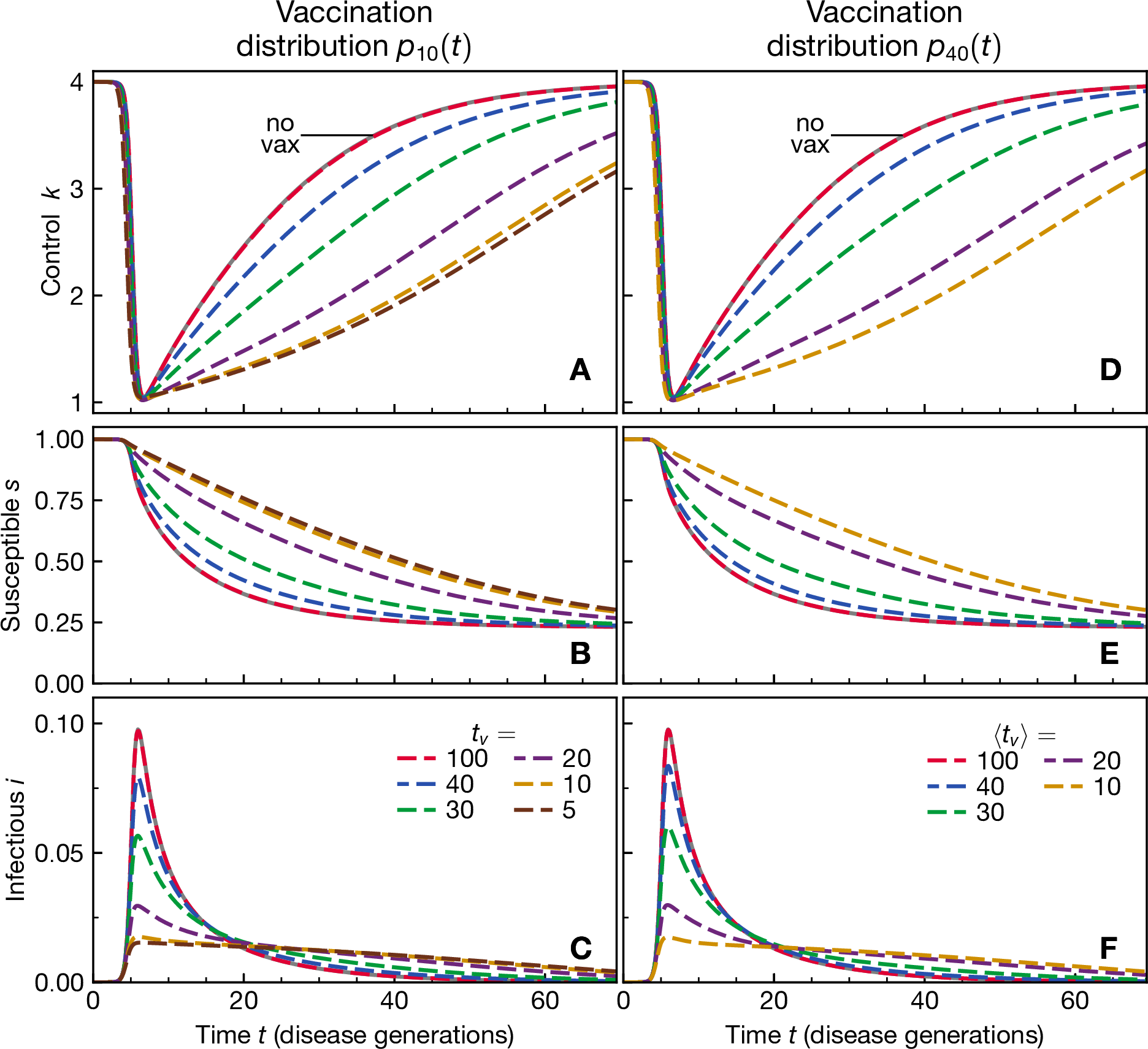

Supplement: S2 Fig — A-C) Nash equilibrium behaviour and course of the epidemic assuming that the vaccination arrival time is distributed as p = p10(t), see Eq (32), with an expected vaccination time 〈tv〉, see panel C for legend. The behaviour and corresponding course of epidemic arising from the assumption that vaccination will not occur are shown as grey solid lines. This is calculated from the case for precisely known vaccination time tv but with tv → ∞. D-F) Same as A-C but for vaccination time distribution p40, see Eq (32). Other parameters: infection cost α = 400, and no economic discounting, τecon → ∞. (TIF) [file pone.0288963.s002.tif]

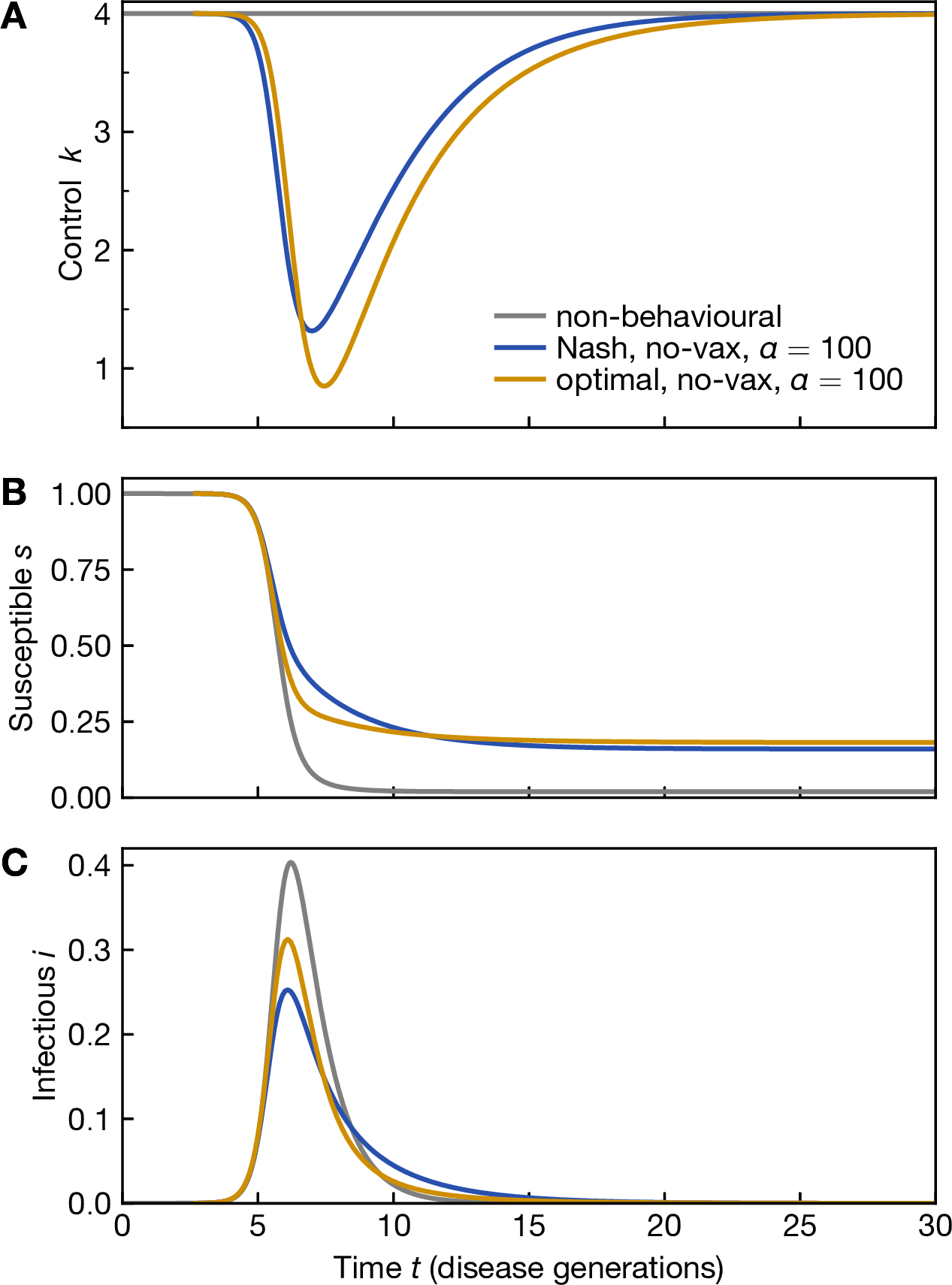

Supplement: S3 Fig — A) Population behaviours during an epidemic: The non-behavioural situation assumes that the pre-epidemic behaviour continues unchanged during the epidemic, k = κ* (blue). In contrast, Nash equilibrium behaviour k without vaccine arrival (black), and optimal behaviour (gold). The corresponding courses of the epidemic are shown in panel B for the susceptibles and panel C for the infected. Note that α = 100, here. (TIF) [file pone.0288963.s003.tif]

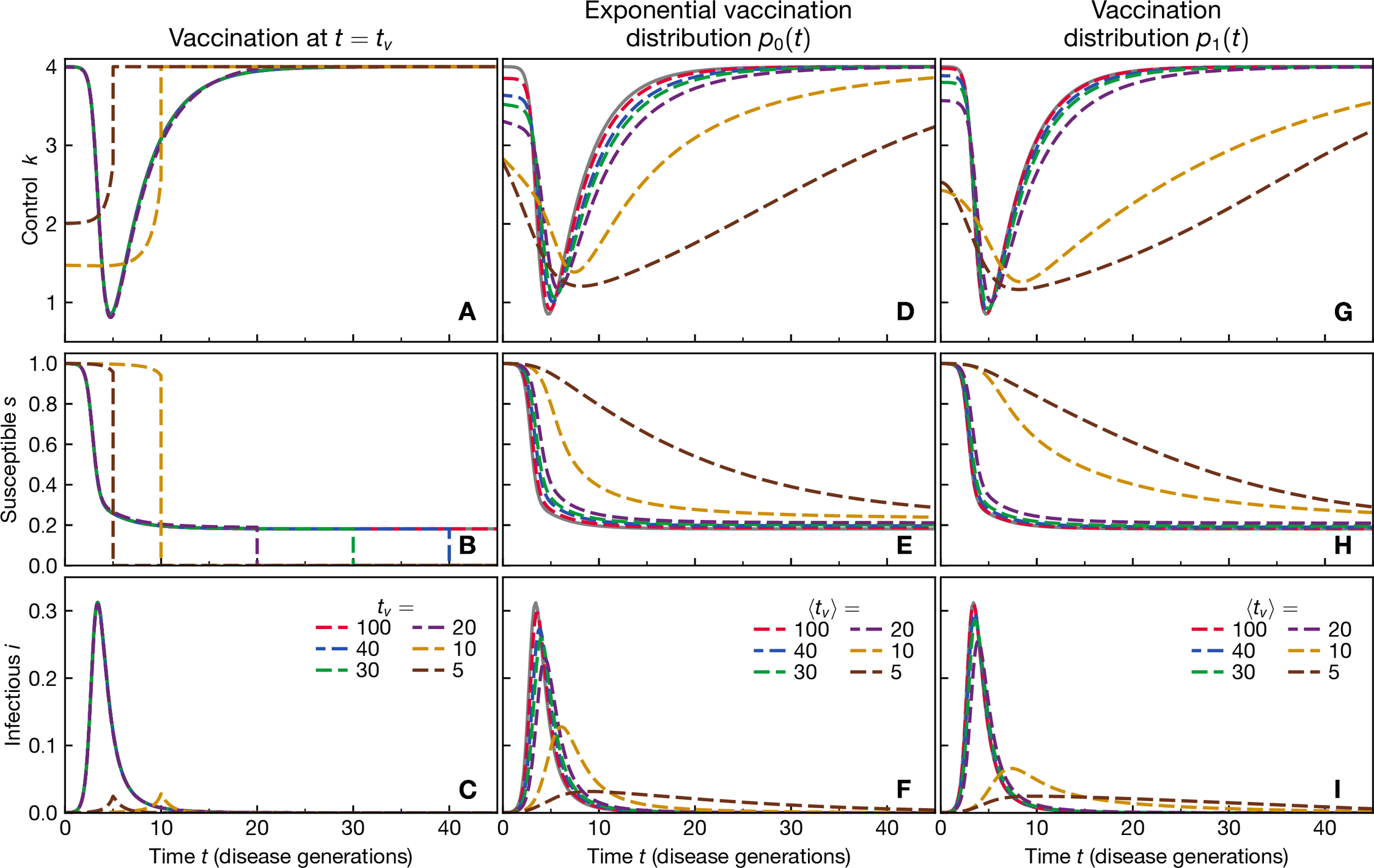

Supplement: S4 Fig — A) Optimal behaviour k for a range of sharp vaccination times tv, as given in the legend of panel C). The behaviour is insensitive to tv for large values and indistinguishable from the behaviour if the vaccination is not expected to occur. When tv is comparable to or shorter than the duration of the epidemic without vaccination, optimal behaviour exhibits strong social distancing; in contrast to the Nash equilibrium, this then already occurs from t = 0 onward. The corresponding courses of the epidemic are shown in panel B) for the susceptibles and C) for the infected. D-F) Optimal behaviour k and course of the epidemic assuming that the vaccination arrival time is exponentially distributed, p = p0(t), see Eq (32), with an expected vaccination time 〈tv〉, see panel F) for legend. The behaviour and corresponding course of epidemic arising from the assumption that vaccination will not occur are shown as gray solid lines. This is calculated from the case for precisely known vaccination time tv but with tv → ∞. G-I) Optimal behaviour k and course of the epidemic assuming that the vaccination arrival time is distributed according to p1, see Eq (32). See panel (i) for legend. Other parameter: infection cost α = 100 and no economic discounting, τecon → ∞. (TIF) [file pone.0288963.s004.tif]
