# Supplementary material for: Rational social distancing in epidemics with uncertain vaccination timing
Source: PLoS One. 2023 Jul 21;18(7):e0288963. doi: 10.1371/journal.pone.0288963 (PMC10361534; doi:10.1371/journal.pone.0288963)
Supplement: S1 File — (PDF) [file pone.0288963.s005.pdf]

Supporting Information: Rational social distancing in epidemics with uncertain vaccination timing

Simon K. Schnyder[1*], John J. Molina[2], Ryoichi Yamamoto[2], Matthew S. Turner[3,4*]

1 Institute of Industrial Science, The University of Tokyo, Tokyo, Japan
2 Department of Chemical Engineering, Kyoto University, Kyoto, Japan
3 Department of Physics, University of Warwick, Coventry, UK
4 Institute for Global Pandemic Planning, University of Warwick, Coventry, UK

* skschnyder@gmail.com (SKS) and m.s.turner@warwick.ac.uk (MST)

Contents

A Utility salvage term for vaccination at a precisely known time **2**

B Some calculations for special choices of vaccination distribution times **3**

C Late time asymptotics **4**
C.1 Utility salvage term from asymptotic solution, Nash equilibrium, without vaccination . . . 6
C.2 Utility salvage term for a distribution of vaccination times . . . 7
C.3 Self-consistency . . . 9

D Rescaling the values or Lagrange multipliers **11**

E Optimal control problem **12**

A Utility salvage term for vaccination at a precisely known time

In case a vaccine becomes available at t_v and immediately protects every susceptible individual, we have $s(t > t_v) = 0$ and $\psi_s(t > t_v) = 0$. The SIR dynamics of eq. (1)

$$\begin{aligned} \dot{s} &= -k\, s\, i \\ \dot{i} &= k\, s\, i - i \\ \dot{r} &= i \end{aligned} \tag{S1}$$

then reduce to

$$\dot{i} = -i \tag{S2}$$

with $r(t)$ relating trivially to $i(t)$. Therefore, the population of infectious $i(t_v) = i_v$ recover as

$$i(t > t_v) = i_v \exp[-(t - t_v)] \tag{S3}$$

and likewise for the individual dynamics

$$\psi_i(t > t_v) = \psi_{i,v} \exp[-(t - t_v)] \tag{S4}$$

Since there is no danger of becoming newly infected after t_v, there is no reason to modify one's behaviour, $\kappa(t) = \kappa^*$. Taking the utility defined by eq. (5)

$$\begin{aligned} U &= \int_0^\infty u(t) dt \text{ with} \\ u &= e^{-t/\tau_{econ}} \left[-\alpha\, \psi_i(t) - \beta \psi_s (\kappa(t) - \kappa^*)^2\right] \end{aligned}$$

we identify the late time utility coming from $t > t_v$ as

$$U_v = -\int_{t_v}^\infty e^{-t/\tau_{econ}} \alpha \psi_i \, \mathrm{d}t \tag{S5}$$

This can be immediately integrated to yield

$$U_v = -\frac{e^{t_v/\tau_{econ}} \alpha \psi_{i,v}}{1/\tau_{econ} + 1} \tag{S6}$$

and the boundary conditions are therefore, with $\psi_{s,v} = \psi_s(t_v)$, $\psi_{i,v} = \psi_i(t_v)$,

$$\begin{aligned} v_s(t_v) &= \frac{\partial U_v}{\partial \psi_{s,v}} = 0 \\ v_i(t_v) &= \frac{\partial U_v}{\partial \psi_{i,v}} = -\frac{-e^{t_v/\tau_{econ}} \alpha}{1/\tau_{econ} + 1} \end{aligned} \tag{S7}$$

Assuming Nash equilibrium, the salvage term reads

$$U_v = -\frac{e^{t_v/\tau_{econ}} \alpha i_v}{1/\tau_{econ} + 1} \tag{S8}$$

B Some calculations for special choices of vaccination distribution times

A relevant class of distributions of the vaccination time is

$$p_n(t) = \frac{t^n}{n!\tau^{n+1}} \exp[-t/\tau] \tag{S9}$$

with integer exponent n and decay time τ. The expectation value of these distributions is

$$\int_0^\infty t p_n(t)\, dt = (n+1)\tau. \tag{S10}$$

The probability distributions can also be written as

$$p_n(t) = \frac{1}{n!\tau^{n+1}} (-1)^n \frac{\partial^n}{\partial\sigma^n} \exp[-\sigma t] \tag{S11}$$

and setting $\sigma = 1/\tau$ afterwards . Introducing the appropriate differential operator O_n, which only acts on σ,

$$O_n = \frac{1}{n!\tau^{n+1}} (-1)^n \frac{\partial^n}{\partial\sigma^n} \tag{S12}$$

we rewrite this

$$p_n(t) = O_n \exp[-\sigma t] \tag{S13}$$

Then the cumulative distribution function can be written as

$$C_n(t) = \int_0^t p_n(t')\, \mathrm{d}t' = O_n \int_0^t \exp[-\sigma t']\, \mathrm{d}t' = O_n \left[\frac{1}{\sigma}(1 - \exp[-\sigma t])\right] \tag{S14}$$

and then setting $\sigma = 1/\tau$. The operator has the convenient property that

$$O_n\left(\frac{1}{\sigma}\right) = \frac{1}{n!\tau^{n+1}} (-1)^n \frac{\partial^n}{\partial\sigma^n} \frac{1}{\sigma} = \frac{1}{(\tau\sigma)^{n+1}} = 1 \tag{S15}$$

for $\sigma = 1/\tau$. With this, we can see that

$$\begin{aligned} C_n(t) &= O_n \left[\frac{1}{\sigma} - \frac{1}{\sigma}\exp[-\sigma t]\right] = 1 - O_n\left[\frac{1}{\sigma}\exp[-\sigma t]\right] \\ &= 1 - \frac{1}{n!\tau^{n+1}} \exp[-\sigma t] \frac{n!}{\sigma^{n+1}} \sum_{l=0}^{n} \frac{\sigma^l t^l}{l!} \\ &= 1 - \exp[-t/\tau] \sum_{l=0}^{n} \frac{t^l}{l!\tau^l} \end{aligned} \tag{S16}$$

Hence, we can write

$$\frac{p_n(t)}{1 - C_n(t)} = \frac{\frac{t^n}{n!\tau^{n+1}} \exp[-t/\tau]}{\exp[-t/\tau] \sum_{l=0}^{n} \frac{t^l}{l!\tau^l}} = \frac{t^n}{n!\tau^{n+1}} \left(\sum_{l=0}^{n} \frac{t^l}{l!\tau^l}\right)^{-1} \tag{S17}$$

which for $t = 0$ evaluates to 0 and for late times tends to

$$\lim_{t\to\infty} \frac{p_n(t)}{1 - C_n(t)} = \frac{1}{\tau} \tag{S18}$$

C Late time asymptotics

Here, we will calculate the asymptotic solution for the population compartments s and i, the individual probabilities of being in said compartments ψ_s and ψ_i, the relevant terms featuring their adjoint values v_i and $(v_s - v_i)$, as well as the Nash equilibrium behaviour κ for late times in order to be able to calculate the salvage term for the utility.

We note that our interest lies in the case where the parameter of the vaccination probability distributions $\sigma = 1/\tau > 0$. This enforces that the distributions have well-defined means.

In the following, we restrict ourselves to vaccination probability distributions as defined by eq. (32). However, the calculations hold for any vaccination distribution $p(t_v)$ which decays exponentially for large t_v.

In order to calculate the asymptotic solution for the population level SIR dynamics for late times, we expand the equations of eq. (S1) according to $s(t) = s_\infty + \delta s(t)$, $i = \delta i(t)$, and $k(t) = \kappa^* - \delta k(t)$, with the δ-quantities assumed to be small and focus on the most important s and i equations to first order in smallness. Rewriting eq. (S1) leads to

$$\begin{aligned} \dot{\delta s} &= -(s_\infty + \delta s)(\kappa^* - \delta k)\delta i \\ \dot{\delta i} &= [(s_\infty + \delta s)(\kappa^* - \delta k) - 1]\delta i \end{aligned} \tag{S19}$$

The necessary boundary conditions are given by $\delta s(t_e) = \delta s_e = s_e - s_\infty$ and $\delta i(t_e) = \delta i_e \equiv i_e$ for some late enough time t_e.

Assuming that the δ-quantities are small, the first line of eq. (S19) immediately reduces to

$$\dot{\delta s} = -s_\infty \kappa^* \delta i \tag{S20}$$

In the second line of eq. (S19), we can drop the terms which are quadratic in δ-quantities

$$\dot{\delta i} = [(s_\infty \kappa^* - 1) + \delta s\, \kappa^* - s_\infty\, \delta k]\delta i, \tag{S21}$$

This equation involves a quantity

$$\eta = 1 - s_\infty \kappa^* \text{ with } 0 < \eta \leq 1, \tag{S22}$$

The case $\eta = 0$ occurs only in the limit $\alpha \to \infty$ and we only consider finite values of α in what follows. In order to confirm that

$$\frac{|\delta s \kappa^* - s_\infty \delta k|}{\eta} \ll 1 \tag{S23}$$

we will later demonstrate in a self-consistent way that δk indeed decays asymptotically to 0 with the same exponent as δi and δs. We numerically confirm that the inequality holds for t_e and its corresponding s_e and i_e for the results presented in this work. Within this assumption, we write

$$\begin{aligned} \dot{\delta s} &= -(1 - \eta)\delta i \\ \dot{\delta i} &= -\eta \delta i \end{aligned} \tag{S24}$$

The SIR variables therefore do not depend on δk to leading order. The differential equations can be integrated to find

$$\begin{aligned} \delta s &= c\frac{1-\eta}{\eta}\exp[-\eta t] \\ \delta i &= c\exp[-\eta t] \end{aligned} \tag{S25}$$

with constant of integration c. Matching conditions at $t = t_e$, written $s(t_e) = s_e$ and $i(t_e) = i_e = \delta i_e$, we have

$$\begin{aligned} s_e - s_\infty \equiv \delta s_e &= c\frac{1-\eta}{\eta}\exp[-\eta t_e] \\ \delta i_e &= c\exp[-\eta t_e] \end{aligned} \tag{S26}$$

We can solve these two equations for the two unknowns c and η in terms of the two "terminal" quantities s_e and i_e. Eliminating c from Eq S26 yields

$$\eta = \frac{1}{\delta s_e/\delta i_e + 1} \tag{S27}$$

Hence $c = i_e \exp[\eta t_e]$ and we can write more simply

$$\begin{aligned} \delta s &= \frac{1-\eta}{\eta} i_e \exp[-\eta(t - t_e)] \\ \delta i &= i_e \exp[-\eta(t - t_e)] \end{aligned} \tag{S28}$$

In order to determine the value of s_∞ (also contained in the definition of η), we evaluate δs at t_e

$$s_e - s_\infty = \frac{1-\eta}{\eta} i_e = \frac{s_\infty \kappa^*}{1 - s_\infty \kappa^*} i_e \tag{S29}$$

and solve this quadratic equation in s_∞,

$$s_\infty = \frac{1 + (s_e + i_e)\kappa^* - \sqrt{[1 + (s_e + i_e)\kappa^*]^2 - 4\kappa^* s_e}}{2\kappa^*} \tag{S30}$$

Requiring that $s_\infty < 1/\kappa^*$, we have selected the negative branch. Hence

$$\eta = \frac{1 - (s_e + i_e)\kappa^* + \sqrt{[1 + (s_e + i_e)\kappa^*]^2 - 4\kappa^* s_e}}{2} \tag{S31}$$

Next, we calculate the asymptotic solution for the individual dynamics. We have $\psi_s(t) = \psi_{s,\infty} + \delta\psi_s(t)$, $\psi_i(t) = \delta\psi_i(t)$, and $\kappa(t) = \kappa^* - \delta\kappa(t)$, assuming that the δ-quantities are all of the same order of smallness. To first order in smallness eq. (2)

$$\begin{aligned} \dot{\psi}_s &= -\kappa\psi_s i \\ \dot{\psi}_i &= \kappa\psi_s i - \psi_i \end{aligned}$$

becomes

$$\begin{aligned} \delta\dot{\psi}_s &= -\psi_{s,\infty}\kappa^*\delta i \\ \delta\dot{\psi}_i &= \psi_{s,\infty}\kappa^*\delta i - \delta\psi_i \end{aligned} \tag{S32}$$

The condition for this expansion to be valid is eq. (S23), the same as for eq. (S24), because in the Nash equilibrium $\psi_s = s$. The "terminal" quantities $\psi_{s,e}$ and $\psi_{i,e}$ can be used as boundary conditions: $\delta\psi_s(t_e) = \psi_{s,e} - \psi_{s,\infty}$ and $\delta\psi_i(t_e) = \psi_{i,e}$. We obtain as solutions

$$\delta\psi_s(t) = \frac{\psi_{s,\infty}\kappa^*}{\eta} i_e e^{-\eta(t-t_e)} \tag{S33}$$

$$\delta\psi_i(t) = \frac{\psi_{s,\infty}}{s_\infty} i_e e^{-\eta(t-t_e)} + \left(\psi_{i,e} - i_e\frac{\psi_{s,\infty}}{s_\infty}\right) e^{-(t-t_e)} \tag{S34}$$

We can determine $\psi_{s,\infty}$ by evaluating eq. (S33) at t_e, i.e. $\psi_{s,e} - \psi_{s,\infty} = \delta\psi_s(t_e)$, and solving for $\psi_{s,\infty}$

$$\psi_{s,\infty} = \psi_{s,e} \frac{\eta}{\eta + i_e \kappa^*} \tag{S35}$$

With this and eq. (S29), we can show that

$$\begin{aligned} \frac{\psi_{s,e}}{\psi_{s,\infty}} &= \frac{\eta + i_e\kappa^*}{\eta} = 1 + \frac{\kappa^*}{1 - s_\infty \kappa^*} i_e = \frac{1}{s_\infty}\left(s_\infty + \frac{s_\infty \kappa^*}{1 - s_\infty\kappa^*} i_e\right) = \frac{s_e}{s_\infty} \\ \Rightarrow \frac{\psi_{s,\infty}}{s_\infty} &= \frac{\psi_{s,e}}{s_e} \end{aligned} \tag{S36}$$

The asymptotic solutions then read

$$\delta\psi_s(t) = \psi_{s,e} \frac{i_e\kappa^*}{\eta + i_e\kappa^*} e^{-\eta(t-tf)} \tag{S37}$$

$$\delta\psi_i(t) = \frac{\psi_{s,e}}{s_e} i_e e^{-\eta(t-tf)} + \left(\psi_{i,e} - i_e \frac{\psi_{s,e}}{s_e}\right) e^{-(t-t_e)} \tag{S38}$$

The complete asymptotic solution is therefore given by eqs. (S28), (S37) and (S38), with definitions eqs. (S22) and (S30).

C.1 Utility salvage term from asymptotic solution, Nash equilibrium, without vaccination

For later reference, we calculate the salvage term arising from integrating out the optimal utility for $t > t_e$ with the asymptotic solution, in the case that no vaccination ever happens. Taking the utility defined by eq. (5)

$$\begin{aligned} U &= \int_0^\infty u(t)dt \text{ with} \\ u &= e^{-t/\tau_{econ}} \left[-\alpha\, \psi_i(t) - \beta\psi_s(\kappa(t) - \kappa^*)^2\right] \end{aligned}$$

we identify the late time utility coming from $t > t_e$ as

$$U_e = -\int_{t_e}^\infty e^{-t/\tau_{econ}} \left[\alpha\delta\psi_i + \beta(\psi_{s,\infty} + \delta\psi_s)\delta\kappa^2\right] dt \tag{S39}$$

We note that in leading order of smallness, the $\delta\kappa^2$ term can be left out and that we therefore have

$$\begin{aligned} U_e &= -\alpha \int_{t_e}^\infty e^{-t'/\tau_{econ}} \delta\psi_i(t')\, dt' \\ \delta\psi i(t') &= \frac{\psi_{s,e}}{s_e} i_e e^{-\eta(t'-t_e)} + \left(\psi_{i,e} - i_e\frac{\psi_{s,e}}{s_e}\right) e^{-(t'-t_e)} \end{aligned} \tag{S40}$$

from eq. (S38) and eq. (S28). Hence

$$\begin{aligned} U_e = &-\alpha \frac{\psi_{s,e}}{s_e} i_e e^{\eta t_e} \int_{t_e}^\infty e^{-(1/\tau_{econ}+\eta)t'}\, dt' \\ &-\alpha\left(\psi_{i,e} - i_e\frac{\psi_{s,e}}{s_e}\right) e^{t_e} \int_{t_e}^\infty e^{-(1/\tau_{econ}+1)t'}\, dt' \end{aligned} \tag{S41}$$

This integrates to

$$U_e = \; - e^{t_e/\tau_{econ}} \alpha \left(\frac{i_e}{1/\tau_{econ} + \eta} \frac{\psi_{s,e}}{s_e} + \frac{1}{1/\tau_{econ} + 1} \left(\psi_{i,e} - i_e \frac{\psi_{s,e}}{s_e} \right) \right) \tag{S42}$$

From this, we can now calculate the boundary conditions for the value equations

$$\begin{aligned} v_s(t_e) &= \frac{\partial U_e}{\partial \psi_{s,e}} = -e^{t_e/\tau_{econ}} \alpha \, \frac{i_e}{s_e} \left(\frac{1}{1/\tau_{econ} + \eta} - \frac{1}{1/\tau_{econ} + 1} \right) \\ v_i(t_e) &= \frac{\partial U_e}{\partial \psi_{i,e}} = -e^{t_e/\tau_{econ}} \alpha \, \frac{1}{1/\tau_{econ} + 1} \end{aligned} \tag{S43}$$

Finally, we are able to assume Nash equilibrium, so that $\psi_{s,e} = s_e$ and $\psi_{i,e} = i_e$. The salvage term then simplifies to

$$U_f = \; - e^{t_e/\tau_{econ}} \alpha \, \frac{i_e}{1/\tau_{econ} + \eta}$$

C.2 Utility salvage term for a distribution of vaccination times

Taking the utility defined by eq. (19)

$$\tilde{U} = \int_0^\infty [1 - C(t)] \, u + p(t) \, U_v(t) \, \mathrm{d}t$$

with u as defined in eq. (5)

$$u = e^{-t/\tau_{econ}} \left[-\alpha \, \psi_i(t) - \beta \psi_s (\kappa(t) - \kappa^*)^2 \right]$$

and U_v as defined in eq. (S6)

$$U_v(t) = -e^{-t/\tau_{econ}} \alpha \, \frac{\psi_i(t)}{1/\tau_{econ} + 1}$$

we identify the late time utility coming from $t > t_e$ as

$$\tilde{U}_e = \int_{t_e}^\infty [1 - C(t)] \, u + p(t) \, U_v(t) \, \mathrm{d}t \tag{S44}$$

Having obtained the asymptotic optimal solution, we are now in the position to explicitly calculate this salvage term. In terms of the small δ-quantities we get

$$\begin{aligned} \tilde{U}_e = \int_{t_e}^\infty \Big[& [1 - C(t)] \, e^{-t/\tau_{econ}} \left[-\alpha \, \delta\psi_i(t) - \beta(\psi_{s,\infty} + \delta\psi_s) \delta\kappa^2 \right] \\ & - p(t) \, e^{-t/\tau_{econ}} \alpha \, \frac{\delta\psi_i(t)}{1/\tau_{econ} + 1} \Big] \, \mathrm{d}t \end{aligned}$$

We note that, to leading order of smallness, the $\delta\kappa^2$ term can be neglected and so

$$\begin{aligned} \tilde{U}_e &= -\alpha \int_{t_e}^\infty e^{-t/\tau_{econ}} \left(1 - C(t) + \frac{p(t)}{1/\tau_{econ} + 1} \right) \delta\psi_i(t) \, \mathrm{d}t \\ \delta\psi_i(t) &= \frac{\psi_{s,e}}{s_e} i_e e^{-\eta(t - t_e)} + \left(\psi_{i,e} - i_e \frac{\psi_{s,e}}{s_e} \right) e^{-(t - t_e)} \end{aligned} \tag{S45}$$

with the asymptotic solution matched to the state of the system at t_e. We can then write

$$\tilde{U}_e = \; - \alpha \frac{\psi_{s,e}}{s_e} i_e M(t_e, \tau_{econ}, \eta, p(t)) - \alpha \left(\psi_{i,e} - i_e \frac{\psi_{s,e}}{s_e} \right) M(t_e, \tau_{econ}, 1, p(t)) \tag{S46}$$

with the function

$$M(t_e, \tau_{econ}, \eta, p(t)) = \int_{t_e}^{\infty} e^{-\eta(t-t_e)} e^{-t/\tau_{econ}} \left(1 - C(t) + \frac{p(t)}{1/\tau_{econ}+1}\right) \mathrm{d}t \tag{S47}$$

introduced for convenience.

Now the boundary conditions for the values can be written as

$$\begin{aligned} v_s(t_e) &= \frac{\partial \tilde{U}_e}{\partial \psi_{s,e}} = -\alpha \frac{i_e}{s_e} \left[M(t_e, \tau_{econ}, \eta, p(t)) - M(t_e, \tau_{econ}, 1, p(t))\right] \\ v_i(t_e) &= \frac{\partial \tilde{U}_e}{\partial \psi_{i,e}} = -\alpha M(t_e, \tau_{econ}, 1, p(t)) \end{aligned} \tag{S48}$$

In the Nash equilibrium, the utility captured by the salvage term can be more simply calculated as

$$\tilde{U}_e = \, -\alpha \, i_e M(t_e, \tau_{econ}, \eta, p(t)). \tag{S49}$$

As before, we choose p and C to be of the form

$$\begin{aligned} p_n(t) &= O_n \exp[-\sigma t] \\ C_n(t) &= O_n \left[\frac{1}{\sigma}(1 - \exp[-\sigma t])\right] \end{aligned} \tag{S50}$$

with $O_n = \frac{1}{n!\tau^{n+1}}(-1)^n \frac{\partial^n}{\partial \sigma^n}$, see eqs. (S12) to (S14), and then setting $\sigma = 1/\tau$ in the end. Focusing on the integral for now and exploiting again that $1 - O_n(1/\sigma) = 0$ when $\sigma = 1/\tau$, we can write

$$\begin{aligned} &M_n(t_e, \tau_{econ}, \eta, \tau) \equiv M(t_e, \tau_{econ}, \eta, p_n(t)) \\ &= e^{\eta t_e} O_n \left[\left(\frac{1}{\sigma} + \frac{1}{1/\tau_{econ}+1}\right) \int_{t_e}^{\infty} e^{-(1/\tau_{econ}+\eta)t} e^{-\sigma t} \, \mathrm{d}t\right] \\ &= e^{-t_e/\tau_{econ}} O_n \left[\frac{1/\tau_{econ}+1+\sigma}{\sigma(1/\tau_{econ}+1)} \frac{e^{-\sigma t_e}}{1/\tau_{econ}+\eta+\sigma}\right] \end{aligned} \tag{S51}$$

Special case: For $n = 0$, we have $O_0 = 1/\tau$ and therefore we find with setting $\sigma = 1/\tau$ that

$$M_0(t_e, \tau_{econ}, \eta, \tau) = e^{-t_e/\tau_{econ}} \left(1 + \frac{1}{\tau(1/\tau_{econ}+1)}\right) \frac{e^{-t_e/\tau}}{1/\tau_{econ}+\eta+1/\tau}$$

and

$$\begin{aligned} \tilde{U}_e = -\, \alpha e^{-t_e/\tau_{econ}} e^{-t_e/\tau} \left(1 + \frac{1}{\tau(1/\tau_{econ}+1)}\right) \times \\ \left[\frac{\frac{\psi_{s,e}}{s_e} i_e}{1/\tau_{econ}+\eta+\frac{1}{\tau}} + \frac{\psi_{i,e} - i_e \frac{\psi_{s,e}}{s_e}}{1/\tau_{econ}+1+\frac{1}{\tau}}\right] \end{aligned} \tag{S52}$$

We see that introducing $\tilde{\alpha} = \alpha(1 + 1/[\tau(1/\tau_{econ}+1)])$ and $1/\tilde{\tau}_{econ} = 1/\tau_{econ} + 1/\tau$ yield

$$\tilde{U}_e = -\tilde{\alpha} e^{-t_e/\tilde{\tau}_{econ}} \left[\frac{\psi_{s,e}}{s_e} i_e \left(\frac{1}{1/\tilde{\tau}_{econ}+\eta}\right) + \left(\psi_{i,e} - i_e \frac{\psi_{s,e}}{s_e}\right) \left(\frac{1}{\tilde{\tau}_{econ}+1}\right)\right] \tag{S53}$$

which is of the same form as eq. (S42), demonstrating that for an exponential distribution of vaccination times, the problem can be restated as one without vaccination but with modified exponential discounting and modified cost of infection.

C.3 Self-consistency

Now we can return to the question of whether we simplified the differential equations appropriately, by examining whether the asymptotic solution is consistent with all assumptions leading to its calculation. Specifically, we need to find criteria for s_e, i_e, t_e which guarantee that the conditions (i) $|\delta s| \ll s_\infty$, (ii) $|\delta k| \ll \kappa^*$ and (iii) $|\delta s \kappa^* - s_\infty \delta k| \ll \eta$ hold for all $t > t_e$, noting that $\delta\kappa = \delta k$ in the Nash equilibrium. In practice, when we solve the optimal control problem on the domain $0 \leq t \leq t_e$ for a certain t_e, we confirm that the three criteria are satisfied by the obtained numerical solution.

Condition (i): Self-consistently, $\delta s(t) \ll s_\infty$ will always hold for large enough times t. We observe that eq. (S29) provides an upper bound for $\delta s(t)$ for all $t > t_e$. Thus we must require that

$$1 \gg \frac{\delta s}{s_\infty} = \frac{s_\infty \kappa^*}{\eta s_\infty} i_e = \frac{\kappa^*}{\eta} i_e \tag{S54}$$

and therefore

$$i_e \ll \frac{\eta(s_e, i_e)}{\kappa^*} \tag{S55}$$

Condition (ii): Some reasoning on whether δk and $\delta\kappa$ are small: In the Nash equilibrium both are given by, see eq. (13),

$$\delta k = \delta\kappa = -\frac{1}{2\beta}\frac{e^{t/\tau_{econ}}}{1 - C(t)}(v_s - v_i) i \tag{S56}$$

From hereon, we only mention δk, with $\delta\kappa$ implied to be equal. Without making any assumptions about v_s and v_i at this point, we have at leading order,

$$\delta k = \frac{1}{2\beta}\frac{e^{t/\tau_{econ}}}{1 - C(t)}(v_s - v_i)\delta i \tag{S57}$$

In order to determine whether δk is of the same order of smallness as δi or not, we need to obtain the asymptotic solution for $(v_s - v_i)$. Since we can obtain an analytic solution for v_i, we discuss it first. The value v_i follows eq. (27)

$$\dot{v}_i = e^{-t/\tau_{econ}} \alpha \left[1 - C(t) + \frac{p(t)}{1/\tau_{econ} + 1}\right] + v_i \tag{S58}$$

In the domain $t_e \leq t < \infty$, the relevant boundary condition is at $t \to \infty$ and corresponds to $\lim_{t\to\infty} v_i(t) = 0$ since there is no corresponding salvage term. The analytic solution for v_i, as defined by eq. (27) can be calculated as follows, if we restrict ourselves to the probability distributions defined as p_n. First we insert the operator forms of p and C, eqs. (S13) and (S14)

$$\dot{v}_i = e^{-t/\tau_{econ}} \alpha \left[1 + O_n\left[\frac{1}{\sigma}(\exp[-\sigma t] - 1)\right] + \frac{O_n \exp[-\sigma t]}{1/\tau_{econ} + 1}\right] + v_i \tag{S59}$$

with

$$O_n = \frac{1}{n!\tau^{n+1}}(-1)^n \frac{\partial^n}{\partial \sigma^n} \tag{S60}$$

We note again that $O_n(1/\sigma) = 1$ when we set $\sigma = 1/\tau$. Therefore we can write

$$\begin{aligned}\dot{v}_i &= v_i + e^{-t/\tau_{econ}} \alpha\, O_n \left[\frac{1}{\sigma}\exp[-\sigma t] + \frac{\exp[-\sigma t]}{1/\tau_{econ} + 1}\right] \\ &= v_i + e^{-t/\tau_{econ}} \alpha\, O_n \left[e^{-\sigma t}\left(\frac{1}{\sigma} + \frac{1}{1/\tau_{econ} + 1}\right)\right]\end{aligned} \tag{S61}$$

Abbreviating with a coefficient A which only depends on parameters τ, σ and τ_{econ}

$$A = \frac{1}{\sigma} + \frac{1}{1/\tau_{econ} + 1} = \frac{1/\tau_{econ} + 1 + \sigma}{\sigma(1/\tau_{econ} + 1)} \tag{S62}$$

we can write more concisely

$$\dot{v}_i = v_i + e^{-t/\tau_{econ}} \alpha\, O_n \left[e^{-\sigma t} A\right] \tag{S63}$$

We can make an ansatz with an integrating factor $g(t)$

$$\frac{\partial}{\partial t}[v_i g] = \dot{v}_i g + \dot{g} v_i = g e^{-t/\tau_{econ}} \alpha\, O_n \left[e^{-\sigma t} A\right] \tag{S64}$$

such that

$$\dot{v}_i + \frac{\dot{g}}{g} v_i = e^{-t/\tau_{econ}} \alpha\, O_n \left[e^{-\sigma t} A\right] \tag{S65}$$

Therefore $\dot{g}/g = -1$ from which we conclude

$$g = e^{-t} \tag{S66}$$

Returning to the original ansatz

$$\frac{\partial}{\partial t}[v_i e^{-t}] = e^{-t} e^{-t/\tau_{econ}} \alpha\, O_n \left[e^{-\sigma t} A\right] \tag{S67}$$

We can integrate this to

$$v_i e^{-t} = \alpha\, O_n \int_{\infty}^{t} e^{-t'} e^{t'/\tau_{econ}} e^{-\sigma t'} A \,\mathrm{d}t' = -\alpha\, O_n \left[\frac{e^{-t} e^{-t/\tau_{econ}} e^{-\sigma t} A}{1 + 1/\tau_{econ} + \sigma}\right] \tag{S68}$$

For the definite integral as written the constant of integration vanishes, since we know that $v_i(t) \to 0$ for $t \to \infty$. Therefore we have the analytic solution for the value of being infectious (after reinserting A)

$$v_i = -\alpha\, e^{-t/\tau_{econ}} O_n \left[\frac{e^{-\sigma t}}{\sigma(1/\tau_{econ} + 1)}\right] \tag{S69}$$

This equation holds even in the limit of $\tau_{econ} \to \infty$ because our interest lies in the case where $\sigma = 1/\tau > 0$. Asymptotically for large times, we observe that

$$v_i(t) \sim e^{-(1/\tau_{econ} + 1/\tau)t} \tag{S70}$$

Next, we calculate an asymptotic solution for $\Delta v \equiv v_s - v_i$, using eq. (27)

$$\begin{aligned} \dot{\Delta v} &= \dot{v}_s - \dot{v}_i \\ &= \frac{1 - C(t)}{e^{t/\tau_{econ}}} \beta \delta\kappa^2 + \Delta v(\kappa^* - \delta\kappa)\delta i - e^{-t/\tau_{econ}} \alpha \left[1 - C(t) + \frac{p(t)}{1/\tau_{econ} + 1}\right] - v_i \end{aligned} \tag{S71}$$

In the domain $t_e \leq t < \infty$, the relevant boundary condition on v_s and v_i is at $t \to \infty$ and corresponds to $\lim_{t\to\infty} v_s(t) = \lim_{t\to\infty} v_i(t) = 0$ since there is no corresponding salvage term. Therefore $\lim_{t\to\infty} \Delta v(t) = 0$. Inserting eq. (S57) for $\delta\kappa$, we obtain

$$\dot{\Delta v} = \Delta v \delta i \left(\kappa^* + \frac{1}{2\beta} \frac{e^{t/\tau_{econ}}}{1 - C(t)} \Delta v \delta i\right) - e^{-t/\tau_{econ}} \alpha \left[1 - C(t) + \frac{p(t)}{1/\tau_{econ} + 1}\right] - v_i \tag{S72}$$

By referring to eqs. (S9) and (S16), we find that asymptotically

$$e^{-t/\tau_{econ}}\alpha\left[1-C(t)+\frac{p(t)}{1/\tau_{econ}+1}\right]\sim e^{-t/\tau_{econ}}e^{-t/\tau} \tag{S73}$$

which is the same behaviour as exhibited by v_i, see eq. (S70). Since asymptotically the exponential terms will dominate, we can conclude, by using eq. (S28),

$$\dot{\Delta v}\sim\Delta v e^{-\eta t}\left(\kappa^*+e^{(1/\tau_{econ}+1/\tau-\eta)t}\Delta v\right)-e^{-(1/\tau_{econ}+1/\tau)t} \tag{S74}$$

To satisfy this equation, Δv has to behave as

$$\dot{\Delta v}\sim\Delta v\sim e^{-(1/\tau_{econ}+1/\tau)t} \tag{S75}$$

since then

$$\begin{aligned}&e^{-(1/\tau_{econ}+1/\tau)t}\\ \sim\ &e^{-(1/\tau_{econ}+1/\tau)t}e^{-\eta t}\left(\kappa^*+e^{(1/\tau_{econ}+1/\tau-\eta)t}e^{-(1/\tau_{econ}+1/\tau)t}\right)\\ &-e^{-(1/\tau_{econ}+1/\tau)t}\\ \sim\ &e^{-(1/\tau_{econ}+1/\tau)t}[e^{-\eta t}\left(\kappa^*+e^{-\eta t}\right)-1]\end{aligned} \tag{S76}$$

which is satisfied for late times at which $\exp[-\eta t]$ can be considered small. Inserting this result into the expression for δk, eq. (S57), and using eqs. (S9), (S16) and (S28) we obtain

$$\delta k\sim\frac{e^{t/\tau_{econ}}}{e^{-t/\tau}}e^{-(1/\tau_{econ}+1/\tau)t}e^{-\eta t}\sim e^{-\eta t} \tag{S77}$$

Thus δk is asymptotically of comparable smallness to δi and δs. As a concrete criterion, we numerically confirmed that $\delta k(t_e)\ll\kappa^*$ holds for all our numerically obtained results.

Condition (iii): For the simplification of eq. (S21) into eq. (S24) to hold, we must require that

$$\frac{|\delta s\,\kappa^*-s_\infty\,\delta\kappa|}{|s_\infty\kappa^*-1|}\ll 1 \tag{S78}$$

Having confirmed that δs and $\delta\kappa=\delta k$ eventually become smaller than any given positive bound, it suffices to test for this after we have numerically solved the control problem.

D Rescaling the values or Lagrange multipliers

In eq. (30), we observe that the term $e^{t/\tau_{econ}}/(1-C(t))$ diverges for $t\to\infty$ with the divergence being compensated by the values. Numerically, this is challenging to handle. Similarly to how it is common practice to introduce the current value Hamiltonian and to absorb the economic discounting into its corresponding current-values, we absorb the diverging terms into the values. We define

$$\hat{v}_s=\frac{e^{t/\tau_{econ}}}{1-C(t)}v_s,\quad \hat{v}_i=\frac{e^{t/\tau_{econ}}}{1-C(t)}v_i \tag{S79}$$

and obtain differential equations for them using eq. (27)

$$\begin{aligned}\frac{\partial}{\partial t}\hat{v}_s&=\left(\frac{1}{\tau_{econ}}+\frac{p(t)}{1-C(t)}\right)\hat{v}_s+\beta(\kappa-\kappa^*)^2+(\hat{v}_s-\hat{v}_i)\kappa i\\ \frac{\partial}{\partial t}\hat{v}_i&=\left(\frac{1}{\tau_{econ}}+\frac{p(t)}{1-C(t)}\right)\hat{v}_i+\hat{v}_i+\alpha\left[1+\frac{p(t)}{1-C(t)}\frac{\tau_{econ}}{1+\tau_{econ}}\right]\end{aligned} \tag{S80}$$

with boundary conditions

$$\begin{aligned}\hat{v}_s(t_e) = & -\alpha\frac{i_e}{s_e}\left[\hat{M}(t_e,\tau_{econ},\eta,p) - \hat{M}(t_e,\tau_{econ},1,p)\right] \\ \hat{v}_i(t_e) = & -\alpha\hat{M}(t_e,\tau_{econ},1,p)\end{aligned} \tag{S81}$$

with helper function

$$\hat{M}(t_e,\tau_{econ},\eta,p) = \frac{e^{t_e/\tau_{econ}}e^{\eta t_e}}{1-C(t_e)}\int_{t_e}^{\infty} e^{-\eta t}e^{-t/\tau_{econ}}\left(1 - C(t) + \frac{p(t)}{1/\tau_{econ}+1}\right)\mathrm{d}t$$

Then we can rewrite eq. (30) to read more simply

$$\tilde{\kappa} = \kappa^* - \frac{1}{2\beta}(\hat{v}_s - \hat{v}_i)i \tag{S82}$$

E Optimal control problem

For a known vaccination time t_v, the optimal control problem corresponding to the social optimum is given by the control that directly optimises the following population level utility,

$$U_p = \int_0^{t_v} u_p(t)dt + U_{p,v} \tag{S83}$$

$$u_p = e^{-t/\tau_{econ}}\left[-\alpha\, i - \beta\, s(k-\kappa^*)^2\right] \tag{S84}$$

$$U_{p,v} = -\frac{e^{-t_v/\tau_{econ}}\alpha\, i_v}{1/\tau_{econ}+1} \tag{S85}$$

such that the SIR equations are satisfied. Then, exploiting Pontryagin's maximum principle, we use the Hamiltonian of the system

$$H_p = -\,e^{-t/\tau_{econ}}\left[\alpha i + \beta\, s(k-\kappa^*)^2\right] - (v_s - v_i)ksi - v_i i \tag{S86}$$

to calculate the dynamics of the adjoint values, which also represent the expected present values of being in states s and i, as

$$\begin{aligned}\dot{v}_s = -\frac{\partial H_p}{\partial s} &= e^{-t/\tau_{econ}}\beta(k-\kappa^*)^2 + (v_s - v_i)ki \\ \dot{v}_i = -\frac{\partial H_p}{\partial i} &= e^{-t/\tau_{econ}}\alpha + (v_s - v_i)ks + v_i\end{aligned} \tag{S87}$$

with boundary conditions

$$v_s(t_v) = 0,\ v_i(t_v) = -\frac{e^{-t_v/\tau_{econ}}\alpha}{1/\tau_{econ}+1} \tag{S88}$$

The optimal control function is unchanged from the equilibrium: From

$$0 = \frac{\partial H_p}{\partial k} = -e^{-t/\tau_{econ}}\left[2\beta s(k-\kappa^*)\right] - (v_s - v_i)si \tag{S89}$$

it follows that

$$k = \kappa^* - \frac{e^{t/\tau_{econ}}}{2\beta}(v_s - v_i)i. \tag{S90}$$

We calculated the social optimum as the numerical solution of eqs. (S1), (S87), (S88) and (S90) via a standard forward-backward sweep approach [1], for $\alpha = 100$ and no vaccination for comparison with the non-behavioral outcome of the epidemic and the Nash equilibrium outcome, in fig. S3. In addition, we calculated the social optimum for a range of vaccination times t_v, see fig. S4 A-C).

If the vaccination arrival follows a probability distribution, we obtain the averaged utility, in analogy to individual decision-making under uncertainty,

$$\tilde{U}_p(k) = \int_0^\infty [1 - C(t)]\, u_p(k(t)) + p(t)\, U_{p,v}(t)\, \mathrm{d}t. \tag{S91}$$

which we also truncate at an end time t_e with a salvage term U_e representing the contribution to the utility from the course of the epidemic after t_e

$$\tilde{U}_p = \int_0^{t_e} [1 - C(t)]\, u_p(k(t)) + p(t)\, U_{p,v}(t) dt + \tilde{U}_{p,e} \tag{S92}$$

$$\begin{aligned} \tilde{U}_{p,e} &= \int_{t_e}^\infty [1 - C(t)]\, u_p(k(t)) + p(t)\, U_{p,v}(t)\, \mathrm{d}t \\ &= -\alpha i_e M(t_e, \tau_{econ}, \eta, p(t)) \end{aligned} \tag{S93}$$

as abbreviated with helper function M, see eq. (S47) and $\eta = \eta(s_e, i_e)$, see eq. (S27). The calculation of $\tilde{U}_{p,e}$ follows in complete analogy to the individual case discussed in SI section C. The (present value) Hamiltonian is

$$\tilde{H}_p = [1 - C]\, u_p + p\, U_{p,v} + v_s(-ksi) + v_i(ksi - i) \tag{S94}$$

Inserting our typical utility, and assuming perfect vaccination, we get

$$\begin{aligned} \tilde{H}_p = &-[1 - C(t)]\, e^{-t/\tau_{econ}} [\alpha i(t) + \beta s(k(t) - \kappa^*)^2] \\ &- p(t)\, e^{-t/\tau_{econ}} \frac{\alpha i(t)}{1/\tau_{econ} + 1} + v_s(-ksi) + v_i(ksi - i) \end{aligned} \tag{S95}$$

and adjoint values defined by

$$\begin{aligned} \dot{v}_s &= -\frac{\partial \tilde{H}_p}{\partial s} = [1 - C(t)] e^{-t/\tau_{econ}} \beta (k(t) - \kappa^*)^2 + (v_s - v_i) ki \\ \dot{v}_i &= -\frac{\partial \tilde{H}_p}{\partial i} = \left[1 - C(t) + \frac{p(t)}{1/\tau_{econ} + 1}\right] e^{-t/\tau_{econ}} \alpha + (v_s - v_i) ks + v_i \end{aligned} \tag{S96}$$

with boundary conditions

$$\begin{aligned} v_s(t_e) &= \frac{\partial \tilde{U}_{p,e}}{\partial s_e} = 0 \\ v_i(t_e) &= \frac{\partial \tilde{U}_{p,e}}{\partial i_e} = -\alpha M(t_e, \tau_{econ}, \eta, p(t)) \end{aligned} \tag{S97}$$

The optimal control follows from

$$0 = \frac{\partial \tilde{H}_p}{\partial k} = -[1 - C(t)] e^{-t/\tau_{econ}} 2\beta s(k - \kappa^*) - (v_s - v_i) is$$

which we solve for k,

$$\kappa = \kappa^* - \frac{1}{2\beta} \frac{e^{t/\tau_{econ}}}{1 - C(t)} (v_s - v_i) i \tag{S98}$$

The result is well defined as long as $1 - C(t) < 0$, i.e. as long as vaccination is not certain to have happened, in which case $k = \kappa^*$. We calculated the social optimum as the numerical solution of eqs. (S1) and (S96) to (S98) via a standard forward-backward sweep approach [1], for $\alpha = 100$ for vaccination arrival distributions p_0 and p_1, see fig. S4 D-I).

References

1. Lenhart S, Workman J. Optimal Control Applied to Biological Models. Chapman and Hall/CRC; 2007.
