# Supplementary material for: Rational social distancing in epidemics with uncertain vaccination timing
Source: PLoS One. 2023 Jul 21;18(7):e0288963. doi: 10.1371/journal.pone.0288963 (PMC10361534; doi:10.1371/journal.pone.0288963)
Supplement: Attachment — Submitted filename: rebuttal.pdf [file pone.0288963.s006.pdf]

Response to the Journal Requirements and to the reviewers

Journal Requirements:

When submitting your revision, we need you to address these additional requirements.

1. Please ensure that your manuscript meets PLOS ONE's style requirements, including those for file naming. The PLOS ONE style templates can be found at https://journals.plos.org/plosone/s/file?id=wjVg/PLOSOne_formatting_sample_main_body.pdf and https://journals.plos.org/plosone/s/file?id=ba62/PLOSOne_formatting_sample_title_authors_affiliations.pdf

Reply:

We believe to have addressed all style requirements with this resubmission.

2. Thank you for stating the following in the Acknowledgments Section of your manuscript:

   "[…]"

We note that you have provided funding information that is not currently declared in your Funding Statement. However, funding information should not appear in the Acknowledgments section or other areas of your manuscript. We will only publish funding information present in the Funding Statement section of the online submission form.

Please remove any funding-related text from the manuscript and let us know how you would like to update your Funding Statement. Currently, your Funding Statement reads as follows:

   "[…]"

Please include your amended statements within your cover letter; we will change the online submission form on your behalf.

Reply:

We apologize for the inconsistencies. Please amend the funding information to read:

"This work was supported by the Grants-in-Aid for Scientific Research (JSPS KAKENHI) under Grants No. 20H00129 (RY), 20K03786 (JJM), 20H05619 (RY), 22H04841 (SKS), the JSPS Core-to-Core Program "Advanced core-to-core network for the physics of self-organizing active matter" (all of us), and the SPIRITS 2020 grant of Kyoto University (JJM). MST acknowledges the generous support of visiting fellowships from JSPS Fellowship, ID L19547, the Leverhulme Trust, Ref. IAF-2019-019, and the kind hospitality of the Yamamoto group. (JSPS: https://www.jsps.go.jp/english/ Leverhulme Trust: https://www.leverhulme.ac.uk/)

The funders had no role in study design, data collection and analysis, decision to publish, or preparation of the manuscript."

If it is acceptable to the journal, we would like the sentence with funding body URLs to be excluded.

Furthermore, we would like to note that we now acknowledge in the paper that:

“The numerical code was written in Python. Figures were generated using the Matplotlib [62] Python library.”

3. In your Data Availability statement, you have not specified where the minimal data set underlying the results described in your manuscript can be found. PLOS defines a study's minimal data set as the underlying data used to reach the conclusions drawn in the manuscript and any additional data required to replicate the reported study findings in their entirety. All PLOS journals require that the minimal data set be made fully available. For more information about our data policy, please see http://journals.plos.org/plosone/s/data-availability.

"Upon re-submitting your revised manuscript, please upload your study’s minimal underlying data set as either Supporting Information files or to a stable, public repository and include the relevant URLs, DOIs, or accession numbers within your revised cover letter. For a list of acceptable repositories, please see http://journals.plos.org/plosone/s/data-availability#loc-recommended-repositories. Any potentially identifying patient information must be fully anonymized.

Important: If there are ethical or legal restrictions to sharing your data publicly, please explain these restrictions in detail. Please see our guidelines for more information on what we consider unacceptable restrictions to publicly sharing data: http://journals.plos.org/plosone/s/data-availability#loc-unacceptable-data-access-restrictions. Note that it is not acceptable for the authors to be the sole named individuals responsible for ensuring data access.

We will update your Data Availability statement to reflect the information you provide in your cover letter.

Reply:
We would like to change our Data Availability statement to read “All relevant data are within the manuscript and its Supporting Information files”. We believe that it will therefore not be necessary to upload any data to a repository.

4. We note that you have stated that you will provide repository information for your data at acceptance. Should your manuscript be accepted for publication, we will hold it until you provide the relevant accession numbers or DOIs necessary to access your data. If you wish to make changes to your Data Availability statement, please describe these changes in your cover letter and we will update your Data Availability statement to reflect the information you provide.

Reply:
Given that we share all our data directly in the paper and SI, we believe this will not be needed.

Additional Editor Comments:
The manuscript has been reviewed by two reviewers and myself. Both reviewers and myself see a

value in the manuscript. The reviewers make several suggestions which I encourage the authors to incorporate in their revision.
The manuscript has been reviewed by two reviewers and myself. Both reviewers and myself see a value in the manuscript. The reviewers make several suggestions which I encourage the authors to incorporate in their revision.

[Note: HTML markup is below. Please do not edit.]

Reviewers' comments:

Reviewer's Responses to Questions

Comments to the Author

1. Is the manuscript technically sound, and do the data support the conclusions?

The manuscript must describe a technically sound piece of scientific research with data that supports the conclusions. Experiments must have been conducted rigorously, with appropriate controls, replication, and sample sizes. The conclusions must be drawn appropriately based on the data presented.

Reviewer #1: Yes

Reviewer #2: Partly

2. Has the statistical analysis been performed appropriately and rigorously?

Reviewer #1: Yes

Reviewer #2: N/A

3. Have the authors made all data underlying the findings in their manuscript fully available?

The PLOS Data policy requires authors to make all data underlying the findings described in their manuscript fully available without restriction, with rare exception (please refer to the Data Availability Statement in the manuscript PDF file). The data should be provided as part of the manuscript or its supporting information, or deposited to a public repository. For example, in addition to summary statistics, the data points behind means, medians and variance measures should be available. If there are restrictions on publicly sharing data—e.g. participant privacy or use of data from a third party—those must be specified.

Reviewer #1: Yes

Reviewer #2: No

4. Is the manuscript presented in an intelligible fashion and written in standard English?

PLOS ONE does not copyedit accepted manuscripts, so the language in submitted articles must be clear, correct, and unambiguous. Any typographical or grammatical errors should be corrected at revision, so please note any specific errors here.

Reviewer #1: Yes

Reviewer #2: Yes

5. Review Comments to the Author

Please use the space provided to explain your answers to the questions above. You may also include additional comments for the author, including concerns about dual publication, research ethics, or publication ethics. (Please upload your review as an attachment if it exceeds 20,000 characters)

Reviewer #1: This work seems nice and informative, which is fairly underpinned by a solid theoretical ground. The authors were interested in an individuals' optimal decision making that is affected by the bulk transmission rate considering their attitude whether social-distancing somehow being accounted or ignored, which is substantially influenced by the timing of when a perfect vaccination is introduced (denoted by t_v), from when all remained S individuals would be immediately transferred to R.
They adopted a standard SIR process, and established behavior model concerning Kappa(t). Subsequently, going thru formulating Hamiltonian, the authors drew Nash Equilibrium on behaver; Eq. (13). This is the case of the timing of vaccination; t_v, regarded as deterministic.
In contrast to this, the authors explored the corresponding Kappa, when the timing of t_v being stochastic; Eq. (30).
They deliver quite interesting and intelligible numerical results to capture what their model has contribute; for instance, Fig. 4. With surging an outbreak of I(t), people withhold their activity by reducing the control; k, to minimize the risk of infection. And following to that, if there is none of introducing vaccination (grey dotted), people tend to back to normal in a rapid manner, which inevitably brings a large amount of infected people. Let alone, if people know the exact timing of vaccination without any uncertainty, behavior; k, immediately resumes to the level of pre-outbreak, and infected individuals die out. The cases with uncertainty let people behave deliberately, staying at highly social-distancing (smeller k). As a whole, the results seem quite likely.
In sum, I favor their approach, seemed scientifically robust, and the presented result is impressive. Hence, I positively evaluate the work.

I would like to give a technical question as below.
As abovementioned, they presumed that a vaccination makes all S individuals to be R in a moment. Which is quite ideal, or say rather unrealistic as an assumption. I've fully understood that the authors intended to be only concerned on the timing of vaccination as a stochastic element (bringing uncertainty) not from other elements. Yet, anyone can agree that the efficacy of vaccination is unequivocally imperfect. By either the so-called Effectiveness or Efficiency idea, the uncertainty of vaccination should be accounted. They should discuss on this point, and are expected to deliver the case considering the uncertainty of vaccine efficacy. They should reference to the concept of

Effectiveness and Efficiency for a vaccination with citing relevant literatures; for instance, (i) Sociophysics Approach to Epidemics, Springer, 2021.

Reply:

We thank reviewer #1 for their positive evaluation of our work.

We agree that the efficacy of vaccination is an interesting quantity to be accounted for. Since we have focused on the rational decision making before the arrival of the vaccine, the efficacy of the vaccine would enter our problem via the salvage term. Currently, we calculate the Nash equilibrium behavior for a population on the condition that a perfect vaccine has not arrived, yet. We have relied in the uncertain vaccination case on a result for precisely known vaccination arrival, namely that its salvage term is can be analytically calculated from the exponential decay of the epidemic after vaccination at t_v. We can envision multiple approaches for the case that the vaccine is not fully effective:

(1) The imperfect vaccination event happens exactly once, at a time t_v, with effectiveness *e*. We then would have to consider the aftermath of the vaccination as its own scenario, with the outcome of the vaccination event as starting condition, i.e. with *e***s*(t_v), i(t_v), and calculate the appropriate Nash equilibrium behavior. This is numerically straightforward for a single t_v. However, we note that the approach proposed in this manuscript requires integrating over the utility contribution (the salvage term) coming out of the vaccination aftermath over all possible t_v. We are currently unable to analytically calculate this salvage term. Being unable to do so indicates that for every iteration of our numerical boundary problem solver, we need to calculate the Nash equilibrium vaccination aftermath numerically (which itself requires hundreds of iterations of our solver) for every possible t_v. This is a numerics problem orders of magnitude more computationally costly than when the vaccine is perfect.

(2) The vaccine becomes available at a time t_v but then is used over time in a vaccination game. Such a game has been in itself the focus of many studies and the task of calculating the appropriate salvage term from it strikes us as even more complex as approach (1).

(3) We drop the condition that the vaccination arrival has not yet occurred when we calculate the Nash equilibrium behavior. This would result in behavior that averages over pre-vaccination situations and post-vaccination situations in which not everyone gained immunity from the vaccine. We believe that this approach confuses the situation.

As a result, we believe that this problem — especially approaches (1-2) — unfortunately lies outside the scope of the present work and best addressed in a future study, while approach (3) would yield less valuable predictions. We believe that the work will be clearer and more concise if we focus our attention on the uncertainty of the vaccination arrival only.

We do agree with the reviewer that we could have cited more about the idea of effectiveness or efficacy of vaccines. Therefore, we now cite and discuss their suggested as well as other additional references.

Reviewer #2: I can evaluate this work can be published on PLOSE.

Reply:
We thank reviewer #2 for their positive evaluation of our work.

Yet, to improve the contents ensuring more impressive information to the audience of PLOSE one, I would like to give following point to be revised in the final MS.

##1. The current form of introduction part is too narrow, some related works are not considered here. Authors can read intervention game related works by following some previous researches:

How quarantine and social distancing policy can suppress the outbreak of novel coronavirus in developing or under poverty level countries: a mathematical and statistical analysis, Biometrics & Biostatistics International Journal.
How Evolutionary Game Could Solve the Human Vaccine Dilemma, Chaos, Solitons & Fractals 152, 111459 (2021).
Social distancing as a public-good dilemma for socio-economic cost: An evolutionary game approach, Heliyon, Heliyon 8, e11497 (2022).
A cyclic epidemic vaccination model: Embedding the attitude of individuals toward vaccination into SVIS dynamics through social interactions, Physica A, 581, 126230 (2021).

Reply:
We are thankful to the referee for pointing out that our introduction is too narrow. We have now added relevant citations, roughly doubling the number of references in the paper.

##2. I could not find any substantial discussion that can fully reflect the assumption of PGG model setup (for example pairwise game). Is it possible to express current model by using pairwise (two by two) evolutionary game mode? Please also mention clearly about all formulation and parameter settings including assumed values that can be fully understandable. (Equation 2)

Reply:
We believe that there are strong parallels of our model to public goods games, in the sense that social distancing is invested by an individual to protect their and, indirectly, the population's health. Also, our current model exhibits a Nash equilibrium and individuals could be incentivized, for instance to target the social optimum. These aspects are discussed by us in the paper, but in response to both reviewer's feedback, we have worked to have a deeper introduction into the field. For instance, we now write more clearly about the social dilemma present in these kinds of problems. At the same time it does not seem to us that our model

matches a social goods game exactly.
As far as we understand it, the framework of a pairwise evolutionary game makes more sense to be applied in situations where the epidemic repeats over many seasons, e.g. as done in [Kabir, Chaos, Solitons & Fractals 152, 111459 (2021)]. It would be possible to extend our framework to this situation in principle, but the current work focuses on a single epidemic season with no loss of immunity. Furthermore, in contrast to an individual facing the binary decision of whether or not to get vaccinated at finite intervals, say at the beginning of each influenza season, our individuals make decisions on a continuous decision space and in continuous time and optimize their actions, while taking the entire course of the epidemic into account.

In our resubmission, we have attempted to be more clear in our introduction of the model and its formulation.

In the previous draft, we had mentioned most parameter values in the main text, $\kappa^* = 4$, $\alpha = 400, \beta = 1, \tau_{econ} \to \infty$, but we had neglected to mention the initial condition of the simulation $i_0 = 3 \cdot 10^{-8}$. We have amended this. For added clarity, we have compiled all parameter values in a single table.

Also, in equation (1), why is there no recover rate or say (recovery rate=1)? Please explain.
I also found some gaps between epidemic model, optimal control model and game model. Author should clearly explore these there different model in the same platform that audience can understood.

Reply:
We had set the units of time such that the recovery rate is 1, by stating that "We measure time in units of the timescale of recovery from an infection". In response to this comment we are now more explicit and add ", i.e. recovery rate = 1."

We had discussed to an extent the distinction between centralised decision making that can give rise to a social optimum and decentralized decision making based on a game that tends to yield a Nash equilibrium due to individuals seeking the optimum of their personal objective function. To address this criticism, we now discuss this in more detail. In addition, we have also calculated the social optimum for key results in an optimal control setting to compare and contrast with the Nash equilibrium outcome. We have added an explanation on how to calculate the optimal control solution in the form of section E in the Supporting Information.

##3. The results seem less impressive and insufficient. I think, it will be very meaningful if authors plot some 2D phase diagram varying two parameters. By introducing 2D heatmap can be explore details explanation of current works.

Reply:

We thank the reviewer for the suggestion. We think the most appropriate quantities to show in a 2D phase diagram would be the expected vaccinations and the peak of the infection curve, both as a function of the expected vaccination time for a range of probability distributions. We had plotted the former for a small selection of distributions in Fig. 3 and the latter can be read off from Figs. 2 and S2. Below we now show this data as two 2D plots. We agree that this adds a new perspective to our analysis, illustrating how the transition from strong to weak social distancing as a function of vaccination time gets sharper, the sharper the expected vaccine arrival distribution is. We use this new figure as Fig. 3 in the new manuscript.

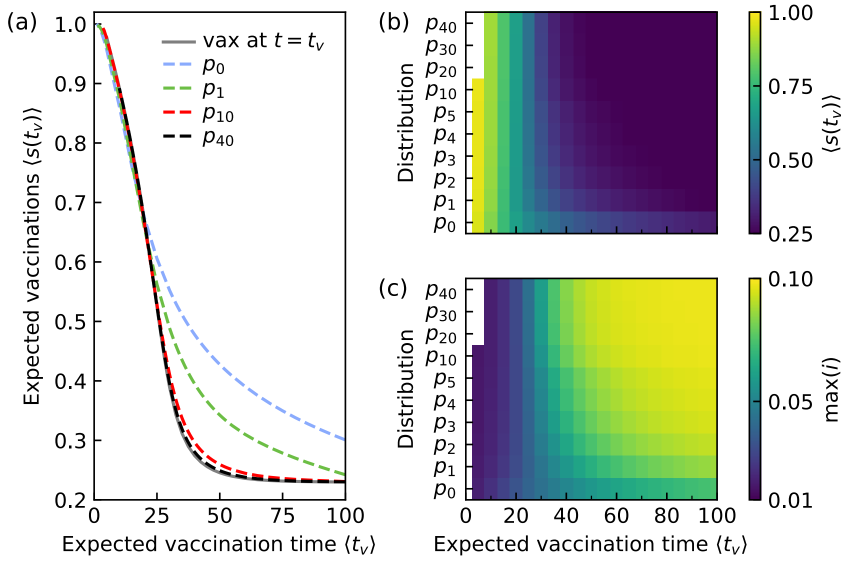

##4. Is dilemma is evolving in this works? If there is some dilemma than please read the following some previous research works and try to introduce here.
Dilemma strength as a framework for advancing evolutionary game theory: "Universal scaling for the dilemma strength in evolutionary games", Physics of Life Reviews 14, 56-58, 2015.
Social efficiency deficit deciphers social dilemmas, scientific reports (Nature), 10, 16092 (2020).
Tanimoto; Evolutionary Games with Sociophysics: Analysis of Traffic Flow and Epidemics, Springer, 2019.
Modelling and analysing the coexistence of dual dilemmas in the proactive vaccination game and retroactive treatment game in epidemic viral dynamics, Proceedings of the Royal Society A 475, 20190484, (2019).

Reply:

We thank the reviewer for pointing out that we should discuss the concept of social dilemma and have amended the text to that effect. There is a social dilemma found in our model, in that

the Nash equilibrium found by the population in a self-organized way has lower utility than the maximum possible social optimum. However, we specifically do not study the type of dilemma that arises in a generational or seasonal vaccination game to which the mentioned references are mostly tailored and focus on a single epidemic, without loss of immunity or a birth process that would inject people into the susceptible compartment. We have added a range of references referring to Social Dilemma in general and the vaccination game in particular.

6. PLOS authors have the option to publish the peer review history of their article (what does this mean?). If published, this will include your full peer review and any attached files.

If you choose "no", your identity will remain anonymous but your review may still be made public.

Do you want your identity to be public for this peer review? For information about this choice, including consent withdrawal, please see our Privacy Policy.

Reviewer #1: No

Reviewer #2: No

Reply:

We are happy to publish the peer review history.
